# Chitosan and Chitosan Nanoparticles Differentially Alleviate Salinity Stress in *Phaseolus vulgaris* L. Plants

**DOI:** 10.3390/plants13030398

**Published:** 2024-01-29

**Authors:** Mekhled M. Alenazi, Aya M. El-Ebidy, Omar A. El-shehaby, Mahmoud F. Seleiman, Khalid J. Aldhuwaib, Heba M. M. Abdel-Aziz

**Affiliations:** 1 Plant Production Department, College of Food and Agriculture Sciences, King Saud University, P.O. Box 2460, Riyadh 11451, Saudi Arabia; 2 Botany Department, Faculty of Science, Mansoura University, Mansoura 35516, Egypt; 3 School of Biological Sciences, University of Reading, Reading RG6 6EX, UK

**Keywords:** antioxidant, chitosan, *Phaseolus vulgaris*, chitosan nanoparticles, abiotic stress

## Abstract

Salinity stress can significantly cause negative impacts on the physiological and biochemical traits of plants and, consequently, a reduction in the yield productivity of crops. Therefore, the current study aimed to investigate the effects of chitosan (Cs) and chitosan nanoparticles (CsNPs) to mitigate salinity stress (i.e., 25, 50, 100, and 200 mM NaCl) and improve pigment fractions, carbohydrates content, ions content, proline, hydrogen peroxide, lipid peroxidation, electrolyte leakage content, and the antioxidant system of *Phaseolus vulgaris* L. grown in clay–sandy soil. Methacrylic acid was used to synthesize CsNPs, with an average size of 40 ± 2 nm. Salinity stress negatively affected yield traits, pigment fractions, and carbohydrate content. However, in plants grown under salt stress, the application of either Cs or CsNPs significantly improved yield, pigment fractions, carbohydrate content, proline, and the antioxidant system, while these treatments reduced hydrogen peroxide, lipid peroxidation, and electrolyte leakage. The positive effects of CsNPs were shown to be more beneficial than Cs when applied exogenously to plants grown under salt stress. In this context, it could be concluded that CsNPs could be used to mitigate salt stress effects on *Phaseolus vulgaris* L. plants grown in saline soils.

## 1. Introduction

Worldwide, agricultural soils are being depleted at an alarming rate due to factors such as abiotic stresses, overpopulation, an increase in natural catastrophes, and alterations in the global climate [1,2]. In arid and semi-arid regions, a part of the cultivable land is being lost due to the phenomenon of soil salinization, which can be caused by a combination of natural factors and human activities. It was estimated that salinity can impact more than 20% of the world’s irrigated land [3,4]. Soil salinity can cause osmotic stress and ionic stress, such as nutritional imbalance, hazardous chemical buildup, and ROS generation. Therefore, salinity stress has become one of the most significant abiotic stressors that can significantly reduce crop yields, particularly for glycophytes (salt-sensitive plants) in comparison to halophytes. In response to these challenges and stresses, plants can develop tolerance mechanisms that are necessary for survival [5]. For instance, the capacity of salt-tolerant plants to compartmentalize Na^+^ and Cl^−^ in vacuoles, preventing their buildup in the cytoplasm or cell walls and avoiding salt toxicity, distinguishes them from salt-sensitive plants [6,7,8].

Salinity can affect plant growth and productivity as a result of limiting leaf-level carbon exchange, root water uptake, and carbohydrate transport in the phloem. Under salt stress conditions, seed germination, root traits, and plant physiological and biochemical traits are negatively inhibited [9,10]. Osmotic stress is considered the first stress that occurs when plants are exposed to soil salinity, and it can immediately affect plant growth and productivity [11,12,13]. However, ion toxicity can occur during plant growth when salt stress touches the threshold, causing negative impacts on plant ion homeostasis and growth [7]. Such a phenomenon has resulted in a focus on using chitosan as a traditional synthetic material or nanoparticle to alleviate the negative effects caused by salt stress, with the main objective of improving crop growth and productivity.

Chitosan (Cs) and chitin are two compounds that have been studied and found to be resistant to abiotic stressors [14,15]. Chitin, made up of N-acetyl glucosamine, is a linear long-chain homopolymer that may take on three different polymorphic forms: α-, β-, and γ-chitin [16,17,18]. After cellulose, chitin is one of the most common biomaterials and may be converted into chitosan by deacetylation. Chitosan, being nontoxic and biocompatible, has gained popularity because of its antibacterial, antioxidant, and chelating characteristics [19,20]. Because it increases nitrogen absorption in plants, chitosan can be a beneficial material for agricultural crop yield and quality [4,21]. The bulk chitosan plays an important role within the plant by activating a number of enzymes in response to various stressors [4]. The epidermis of leaves and stems quickly absorbs chitosan, prolonging the contact period and promoting the absorption of beneficial chemicals [22,23]. However, after being treated with chitosan and its derivatives, the amount of malondialdehyde (MDA) can be decreased, and the membrane damage can be reduced.

Chitosan has the ability to enhance the resistance of different crops to different abiotic stresses through a transduction pathway via secondary messengers [15]. For example, it can enhance photosynthetic rate and stomatal closure via ABA synthesis, stimulate antioxidant enzymes through nitric oxide and hydrogen peroxide signaling pathways, and promote sugars and amino acids that are needed for adjusting the osmotic, stress signaling, and energy metabolism under abiotic stresses [15]. In addition, some biochemical and molecular changes in plant growth, such as callose apposition, increases in cytosolic Ca^2+^ callose apposition, chromatin alterations, inhibition of plasma membrane H^+^-ATPase, phytoregulators, and alkaloid synthesis, were observed with the application of chitosan [24,25]. In this respect, the application of bulk chitosan during salinity stress resulted in an increase in antioxidant enzyme activities and reduced MDA content, which eventually reduced the negative effects of salt stress in *Zea mays* L. [26], *Vigna radiata* L. [27], and *Plantago ovata* L. [28].

As opposed to bulk Cs, chitosan nanoparticles (CsNPs) can be an effective alternative because of their tiny size and their surface and interface effects [29,30]. The beneficial usage of chitosan nanoparticles (CsNPs) in agricultural systems has been reported since they contain some bioactive chemicals [31,32] that can sustain and enhance the mechanisms and biostimulant capacity to mitigate environmental stresses. CsNPs have some advantages, such as a high surface area and low toxicity, which make them a more beneficial option than traditional agrochemicals. Seed germination was increased in salt-sensitive bean plants when CsNPs were applied at 0.1%, 0.2%, and 0.3% alongside 100 mM NaCl [32,33]. Chitosan nanoparticles can enhance apple seedlings that were exposed to abiotic stress by enhancing the activities of antioxidant enzymes such as catalase (CAT) and superoxide dismutase (SOD) and reducing lipid peroxidation [33].

The common bean *(Phaseolus vulgaris* L.) is the most widely consumed legume worldwide. Due to their high protein content and unique blend of carbs, fiber, and minerals (especially iron and zinc), beans are widely known as a remarkable food resource and also beneficial to human health [34,35,36]. However, the common bean is considered a salt-sensitive crop [37], and salinity stress can pose negative impacts on its growth and biomass productivity.

Therefore, the main objective of this study was to investigate the effects of the exogenous application of conventional and nanoparticle forms of chitosan (0.05%) on the growth, yield, and biochemical compounds of common beans grown under salt stress (i.e., 25, 50, 100, and 200 mM NaCl).

## 2. Results

### 2.1. Effects of Cs or CsNPs on Growth and Yield Traits of Common Bean Grown under Salt Stress

When compared to plants that were not treated (i.e., control; Table 1 and Table 2), common bean plants that were given Cs or CsNPs had better growth and yield. Different levels of salinity stress, on the other hand, led to a noticeable reduction in growth and yield, going from S4 to S3 to S2 to S1 to control. When Cs or CsNPs were added to plants that were stressed by salt, the growth and productivity were better for the plants than those obtained from un-stressed plants (Table 1 and Table 2). In salt-stressed common bean plants (i.e., 50 mM NaCl), the application of CsNPs and Cs improved plant height by 38.89 and 13.30%, shoot length by 45.08 and 43.30%, number of seeds per pod by 35.25 and 32.25%, seed weight per pod by 80.95 and 28.57%, and seed yield per plant by 40.80 and 8.69% in comparison to those obtained from untreated plants. Plant water content showed slightly variable changes in all treatments as compared with the control. The summary of the two-way ANOVA of yield traits is shown in Table 3.

### 2.2. Effects of Cs or CsNPs on Photosynthetic Pigments and Total Carbohydrates Contents of Common Bean Grown under Salt Stress

The salinity treatments in the following sequence (S4 > S3 > S2 > S1) showed a significant decrease in the studied photosynthetic pigment traits when compared with those obtained from the untreated plants (i.e., control; Table 4). However, the application of either Cs or CsNPs mitigated the negative effects of salt stress and resulted in an increment in photosynthetic pigments as compared with those obtained from the control (Table 4). Nevertheless, CsNPs showed better beneficial effects than Cs in the mitigation of salt stress and enhancement of the photosynthetic pigment traits. As compared to the control, the following treatment sequence: S4 > S3 > S2 > S1 showed a significant increase in TSS and a significant decrease in starch and total carbohydrate content. Treatment with Cs or CsNPs showed a significant increase in TSS, starch, and total carbohydrates content according to the following sequence: CsNPs > Cs > C. In salt-stressed common bean plants (i.e., 50 mM NaCl), the application of CsNPs and Cs increased total pigments by 14.88 and 10.41%, TSS by 8.34 and 2.31%, starch by 22.44 and 13.53%, and total carbohydrates by 19.28 and 11.02% in comparison to those obtained from untreated plants. Table 5 shows the summary of the two-way ANOVA of photosynthetic pigments and carbohydrate fractions.

### 2.3. Effects of Cs or CsNPs on Ions Contents of Common Bean Grown under Salt Stress

**Figure 1:**
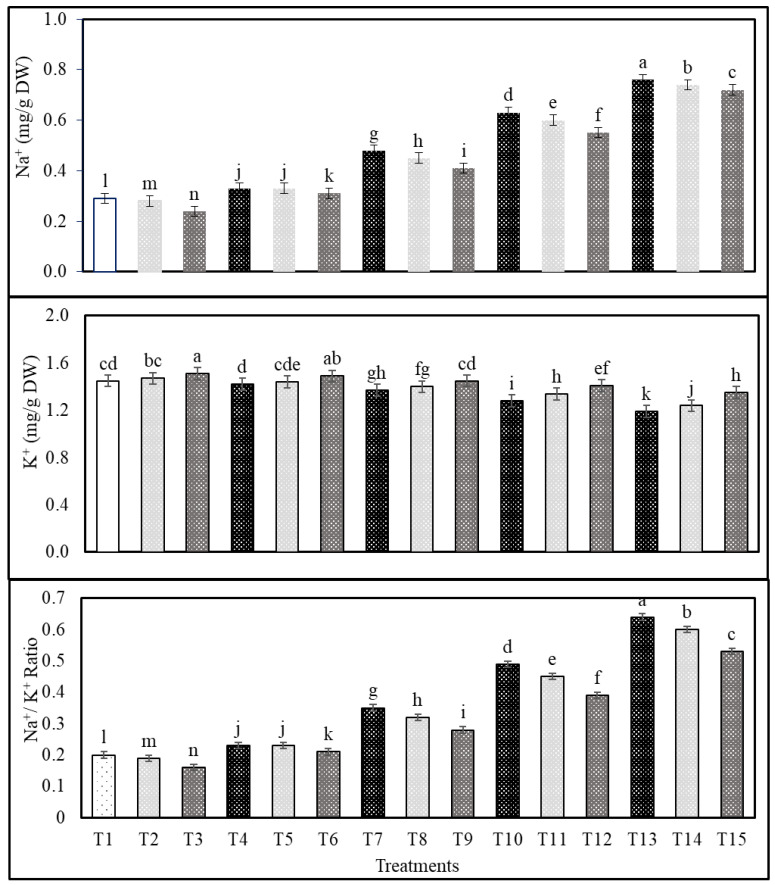
Effects of exogenous application of chitosan or chitosan nanoparticles on Na^+^, K^+^ contents, and Na^+^/K^+^ ratio in shoots of salt-stressed common bean plants at the flowering stage. (T1; control, T2; Cs, T3; CsNPs, T4; (S1), T5; (S1 + Cs), T6; (S1+ CsNPs), T7; (S2), T8; (S2 + Cs), T9; (S2 + CsNPs), T10; (S3), T11; (S3 + Cs), T12; (S3 + CsNPs), T13; (S4), T14; (S4 + Cs), T15; (S4 + CsNPs). Bars = standard error (±SE). Means (of three replicates) denoted by similar letters are not significantly different at *p* ≤ 0.05 using the Duncan test.

**Figure 2:**
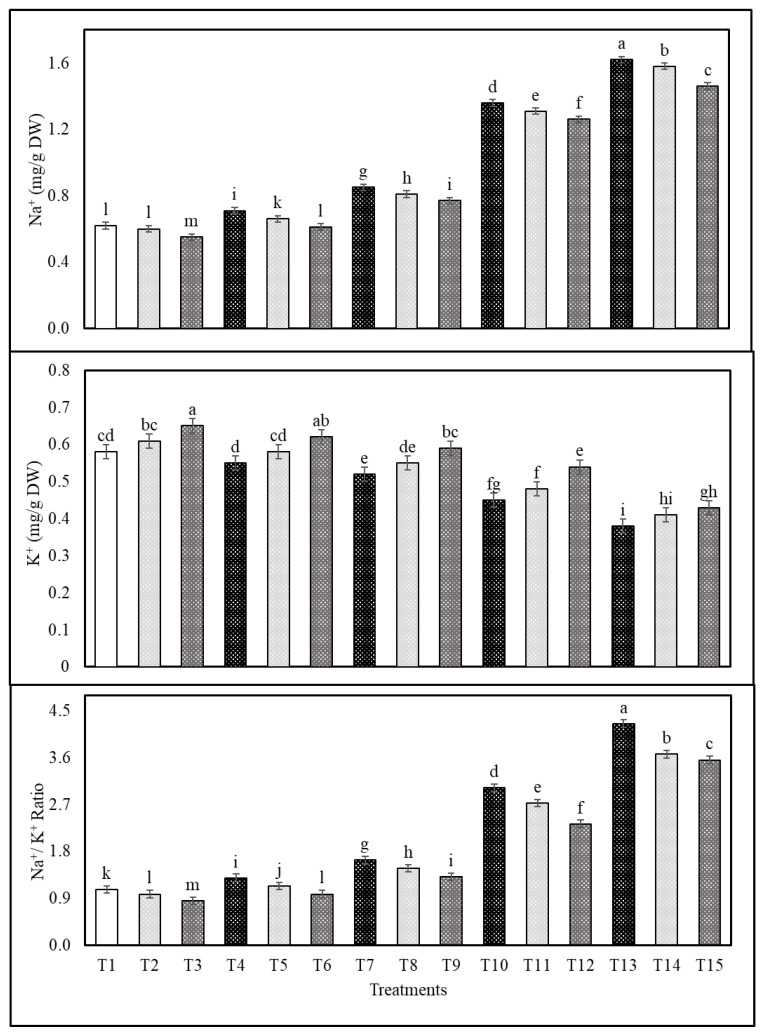
Effects of exogenous application of chitosan or chitosan nanoparticles on Na^+^, K^+^ contents, and the Na^+^/K^+^ ratio in the roots of salt-stressed common bean plants at the flowering stage. (T1; control, T2; Cs, T3; CsNPs, T4; (S1), T5; (S1 + Cs), T6; (S1+ CsNPs), T7; (S2), T8; (S2 + Cs), T9; (S2 + CsNPs), T10; (S3), T11; (S3 + Cs), T12; (S3 + CsNPs), T13; (S4), T14; (S4 + Cs), T15; (S4 + CsNPs). Bars = standard error (±SE). Means (of three replicates) denoted by similar letters are not significantly different at *p* ≤ 0.05 using the Duncan test.

The subsequent sequence of salinity treatments follows: S4 > S3 > S2 > S1 resulted in an increase in the Na^+^ and Na^+^/K^+^ ratio and a decrease in the K^+^ content in shoots and roots of common bean plants, but the exogenous application with Cs or CsNPs mitigated the adverse effects of salt-stress in terms of reducing Na^+^ and the Na^+^/K^+^ ratio and increasing the content of K^+^ compared with those obtained from control (Figure 1 and Figure 2). In salt-stressed common bean plants (i.e., 50 mM NaCl), the application of CsNPs and Cs reduced shoots Na^+^ by 14.58 and 6.25% and the Na^+^/K^+^ ratio by 5.83 and 2.18% and increased shoot K^+^ by 20.00 and 8.57% in comparison to those obtained from untreated plants (Figure 1). Moreover, the application of CsNPs and Cs reduced roots Na^+^ by 9.41 and 4.70% and Na^+^/K^+^ ratio by 13.46 and 5.76%, and increased shoots K^+^ by 19.63 and 9.81% of salt-stressed common bean plants (i.e., 50 mM NaCl) compared to those obtained from untreated plants (Figure 2); data are also available in Supplementary Table S1.

### 2.4. Effects of Cs or CsNPs on Biochemical Traits of Common Bean Grown under Salt Stress

**Figure 3:**
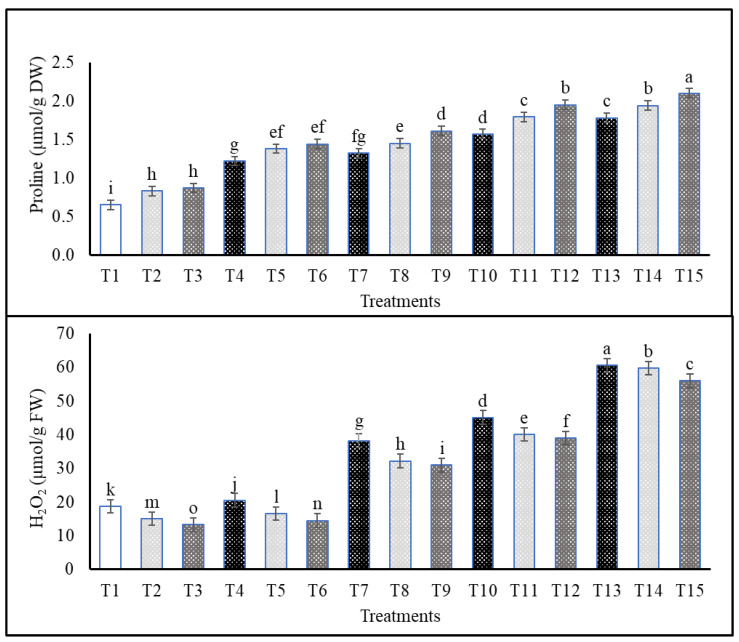
Effects of exogenous application of chitosan or chitosan nanoparticles on the proline and hydrogen peroxide content of salt-stressed common bean plants at the flowering stage grown in clay–sandy soil. (T1; control, T2; Cs, T3; CsNPs, T4; (S1), T5; (S1 + Cs), T6; (S1+ CsNPs), T7; (S2), T8; (S2 + Cs), T9; (S2 + CsNPs), T10; (S3), T11; (S3 + Cs), T12; (S3 + CsNPs), T13; (S4), T14; (S4 + Cs), T15; (S4 + CsNPs). Bars = standard error (±SE). Means (of three replicates) denoted by similar letters are not significantly different at *p* ≤ 0.05 using the Duncan test.

**Figure 4:**
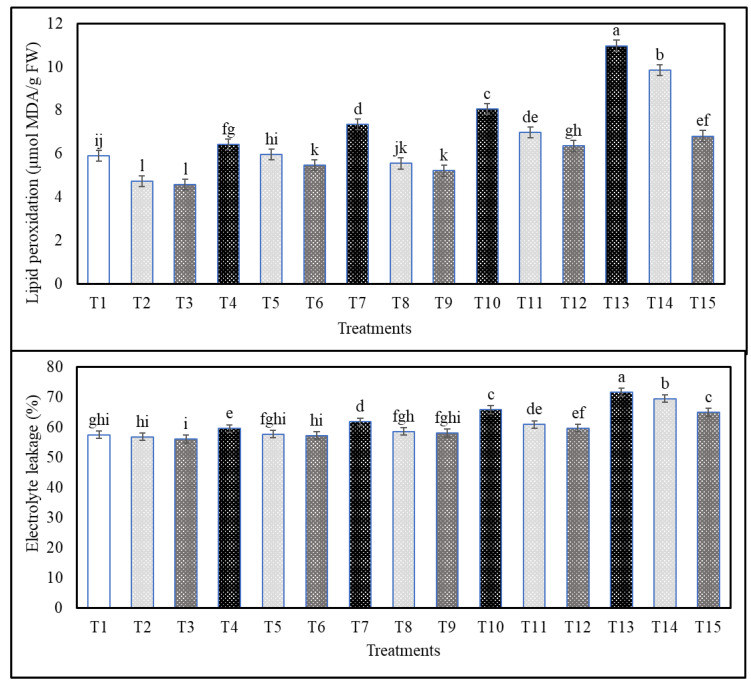
Effects of exogenous application of chitosan or chitosan nanoparticles on the lipid peroxidation and electrolyte leakage content of salt-stressed common bean plants at the flowering stage grown in clay–sandy soil. (T1; control, T2; Cs, T3; CsNPs, T4; (S1), T5; (S1 + Cs), T6; (S1+ CsNPs)**,** T7; (S2), T8; (S2 + Cs), T9; (S2 + CsNPs), T10; (S3), T11; (S3 + Cs), T12; (S3 + CsNPs), T13; (S4), T14; (S4 + Cs), T15; (S4 + CsNPs). Bars = standard error (±SE). Means (of three replicates) denoted by similar letters are not significantly different at *p* ≤ 0.05 using the Duncan test.

As compared with the non-stressed plants, the following sequence of salinity treatments: S4 > S3 > S2 > S1 significantly increased proline content even when plants were treated with Cs or CsNPs (Figure 3). On the other hand, salt-stressed plants treated with CsNPs had higher proline than salt-stressed plants treated with Cs (Figure 3). In comparison to control, plants treated with different salt-stress levels showed the following sequence: S4 > S3 > S2 > S1 for the increment of H_2_O_2_, lipid peroxidation, and electrolyte leakage in plants under different treatments of Cs and CsNPs (Figure 3 and Figure 4). However, stressed plants treated with CsNPs had the lowest values of H_2_O_2_, lipid peroxidation, and electrolyte leakage, followed by plants treated with Cs under each salt-stress level in comparison to those obtained from untreated plants (data are also available in Supplementary Table S2).

### 2.5. Effects of Cs or CsNPs on Antioxidants System in Common Bean Grown under Salt Stress

In general, the following sequence of salinity treatments increased the contents of total phenols, ascorbic acid (AsA), reduced glutathione (GSH), peroxidase (POX), polyphenol oxidase (PPO), ascorbate peroxidase (APX), superoxide dismutase (SOD), and glutathione reductase (GR): S4 > S3 > S2 > S1 except for catalase (CAT) (Table 6). Also, the foliar application with either Cs or CsNPs resulted in an increase in antioxidant activity when compared with those obtained from untreated plants. However, as compared with the individual salinity levels, salt-stressed plants that were treated either with Cs or CsNPs had the highest values of antioxidant activity (Table 6). Under salt stress of S2 (50 mM NaCl), the application of CsNPs and Cs increased CAT by 96.88 and 83.26%, respectively, and SOD by 16.47 and 8.03%, respectively, in comparison to those obtained from untreated plants. A summary of the two-way ANOVA of these data is found in Table 7.

## 3. Discussion

Soil salinity is a serious issue in arid and semi-arid regions because it can affect plant production and growth through complex interaction processes [29,33,38]. According to some investigations and statistics, about 20% of the world’s irrigated land is affected by salinity [5,39]. It has been shown that osmotic effects and oxidative stress are considered responsible for the detrimental effects of salt stress on plant growth [40,41]. Nanoparticles (NPs) can improve morphological characteristics and enhance non-enzymatic and enzymatic antioxidants, stress-related gene expression, and osmolyte production in plants under drought and salt stress conditions [42]. Sustainable agricultural production, decreased nutrient loss, disease control, and enhanced health are considered some of the different benefits that can be associated with the usage of nanomaterials [42,43].

The characterization of the obtained chitosan nanoparticles in this study yielded stable round particles with a mean diameter of 40 ± 2 nm and a zeta potential of +30.10 mV, indicating the cationic nature of the generated nanoparticles. To form CsNPs, methacrylic acid (MAA) is polymerized in the presence of Cs [44,45]. Since Cs molecules in solution are in a cationic electrolytic form, this facilitates the formation of specific structures via electrostatic reactions with methacrylic acid. To prove that, CsNPs can include chemical functional groups. Depending on the conditions under which the spectra were collected (i.e., 400 cm^−^^1^ to 4000 cm^−^^1^), CsNPs might include a wide range of functional groups such as C=O, OH, and COOH. Ionic interaction between MAA and Cs associated with nanoparticle formation is evidenced by the presence of two additional bands, located at 1646 and 1546 cm^−^^1^ and attributed to the COO and NH_3_^+^ groups, respectively. Nanoparticles with poly-MAA in their composition can be identified by two bands at 1741 and 1835 cm^−^^1^ (C=O) [46].

In this study, foliar administration of Cs or CsNPs to common bean plants mitigated the negative effects of salt stress on a number of yield metrics. Water stress negatively impacted agricultural yield and yield components in *Vigna unguiculata* and tomato [47,48]. Such findings are consistent with those obtained by Maggio et al. [49], who reported that the reduction in yield as a result of salinity stress could be attributable to a combination of osmotic stress in the root growing medium due to the relatively high solute concentrations and the unique toxicity that can be caused by the addition of high levels of Na^+^ and Cl^−^ to the plant (tomato), consequently negatively affecting the physiological and biochemical processes that have impeded its growth and development. Chitosan’s efficacy and its role as a signal molecule are cited in [2,50]. To keep the biomass and production of tomato and pepper plants hydrated during times of low water availability, chitosan was used as a foliar spray [48,51]. This decreased the rate of gas exchange and transpiration from the leaves. Due to their higher surface area, better adsorption ability, nontoxicity, and capacity to encapsulate other molecules with high encapsulation efficacy, chitosan nanoparticles may be more effective in salt-stressed plants [31,32,33,34]. This action of chitosan nanoparticles is made more likely by the aforementioned reasons [32]. Positive effects of CsNPs on plant growth parameters, chlorophyll content, and antioxidant enzyme activities may have increased chilli pepper yield in terms of fruit number per plant, average fresh weight of fruit, fruit weight, and total yield [52]. There is little information with regard to the application of Cs or CsNPs before imposing salinity stress on common bean plants. Our study pointed out the effects of such an application on common bean yield metrics and other biochemical aspects of the cells.

In our study, all photosynthetic pigments were lower in the salinity treatments relative to the control but higher in the treatments that received foliar applications of Cs or CsNPs, either alone or in combination with salt stress. Total chlorophyll, chlorophylls a and b, and carotenoids in bitter melon were all reduced by salt stress [53]. Salt stress can have a low impact on photosynthesis due to the destruction of photosynthetic pigments and/or decreased pigment formation. Another explanation for these attenuated results is an increase in the synthesis of proteolytic enzymes such as chlorophyllase, the major source of chlorophyll degradation, and/or damage to the photosynthetic system, as recorded in sunflower and *Avena* species [2,54].

Increased nitrogen, phosphate, and potassium uptake and improved nitrogen transport to leaves may be responsible for the correlation between chitosan concentration and leaf chlorophyll content increase, as recorded in tomatoes [4,55]. Salt’s damaging effects on chilli pepper plants’ photosynthetic pigments are lessened by CsNPs [52]. By scavenging reactive oxygen species (ROS), protecting chlorophyll content and photosynthesis parameters, and improving photosystem functioning, foliar application of polyamines under salinity conditions may mitigate the deleterious effect of salinity on chlorophyll content in *Bakraii citrus* [56].

In our study, total carbohydrate content, excluding total soluble sugars, decreased under salt stress compared to the control but increased after foliar application of Cs or CsNPs. When exposed to salt stress, all wheat varieties experienced a dramatic reduction in total carbs and starch [57]. While the total carbohydrate content of rice shoots progressively and considerably dropped as salt levels increased from 0 to 60 mM NaCl [58], the TSS was dramatically increased compared to those obtained from plants grown in the control treatment.

After observing a decrease in both total carbohydrates and leaf photosynthetic pigments in a salt-stressed rice plant, researchers concluded that salinity may limit photosynthetic activity and/or promote the partial consumption of carbohydrates in other metabolic pathways [58,59]. More soluble sugars were found in safflower leaves when salt stress levels rose [60]. In addition, the enzyme sucrose-phosphatase is stimulated by salt treatments. Plants may be producing more of this enzyme, which might explain the increase in soluble sugars. An increase in soluble carbohydrates is necessary to maintain water balance and osmotic conditions when sodium chloride concentration and ion accumulation in plant tissues grow, as recorded in canola [61]. Wheat chlorophyll and photosynthetic rate were both increased by chitosan treatment while the plant was experiencing salt stress [62,63]. More glucose and total carbohydrates were produced by wheat plants when chitosan nanoparticles were employed than when NaCl was used [63,64]. White clover leaves treated with chitosan nanoparticles exhibit elevated levels of glucose, fructose, mannose, trehalose, sorbitol, and myoinositol, all of which are simple sugars. Many genes involved in carbohydrate transport and metabolism are also upregulated by exposure to other recognized sugars [65]. Nanoparticles and salinity had a synergistic impact that greatly increased concentrations of the amino acid proline and the soluble sugar glucose. Natural solutes like these are crucial for maintaining cellular homeostasis and osmoregulation when exposed to high levels of salt, as observed in Roselle plants [66].

In this study, in contrast to the control group, which showed an increase in Na^+^ and the Na^+^/K^+^ ratio and a decrease in K^+^ content as shown by S4 > S3 > S2 > S1 values, treatment with Cs or CsNPs alone showed a decrease in Na^+^ and the Na^+^/K^+^ ratio and an increase in K^+^ content. In optimum conditions, the cytoplasm of plants contains a lot of K^+^ and not too many Na^+^ ions. Because of the shift in Na^+^ concentration towards the roots during salt stress, K^+^ absorption and the K^+^, Na^+^ balance are both thrown off, as recorded for Australian wild rice [67]. The leaves of the bitter melon show significant changes in Na^+^ and K^+^ concentrations in response to elevated salinity [53]. The osmotic adjustment was thrown off, and the K^+^/Na^+^ ratio dropped as a result of the increased absorption of Na^+^ in wheat [68,69]. The application of chitosan prevented the disturbance of ion homeostasis, which led to a rise in the concentrations of N, P, K, Mg, and Fe and a decrease in the concentrations of Na in plants subjected to salt stress, as recorded in *Moringa oleifera* [70]. Under salt stress, *Moringa peregrina*, which had been treated with nanoparticles, had its nutrient, mineral, and metal content improved [71].

The current study found that the concentration of proline rose in response to salt stress and that this rise was augmented by foliar application of either Cs or CsNPs. It was hypothesized that certain crop species would show the salt-induced increase in proline levels as recorded in mung bean and wheat [38,69]. Sunflower plants irrigated with salt water had significantly increased concentrations of free amino acids, proline, and total soluble solids (TSS). The resilience of cells to salt stress can be increased by increasing osmoprotectants or compatible solutes (free amino acids, proline, and TSS) [2,72]. Since chitosan encourages the accumulation of the amino acid proline, it has the potential to mitigate salt’s adverse effects. Grapevines [70,73], stevia [74], and milk thistle [75] have all been the subjects of similar studies. The buildup of proline is greatly enhanced when nanoparticles are used. Nanoparticle insertion into salt-stressed tomato leaves increased proline synthesis [76]. The results of two separate squash experiments [71,77] were identical.

In our study, treatment with Cs or CsNPs alone or sprayed prior to salinity stress resulted in a reduction in H_2_O_2_, lipid peroxidation, and electrolyte leakage as compared to the control (S4 > S3 > S2 > S1), respectively. Increased electrolyte leakage and increased generation of hydrogen peroxide and malondialdehyde (MDA) were seen in wheat leaves when salt stress was present [69]. Similarly, a lack of non-enzymatic antioxidant molecules (ascorbate, flavonoids, phenolics, and carotenoids) and an increasing cascade of reactive oxygen species (ROS) following exposure to salt can account for the accumulation of H_2_O_2_ and MDA in wheat [69]. Grapevines are particularly vulnerable to the effects of stress, and previous studies have shown that this can lead to membrane damage and increased MDA levels [78].

Since the cell membrane is the primary source of ion-specific damage due to salinity, electrolyte leakage may be utilized as a damage indicator to determine the rate of injury in the cell membrane under salt stress conditions, as recorded in grapevine, lettuce, New Zealand spinach, and common purslane [78,79]. Application of CsNPs derived from chitosan improved membrane integrity in *Catharanthus roseus* by decreasing levels of MDA and H_2_O_2_, indicating decreased lipid peroxidation and oxidative stress. Because of its high concentration of hydroxylated amino groups, chitosan is an effective scavenger of reactive oxygen species (ROS) [41,80].

In our investigation, when compared to the control treatment, different salinity treatments, before the application of Cs or CsNPs, resulted in an increase in all studied antioxidant enzymes except catalase (CAT). Plants respond to stress by activating their antioxidant systems, which include both enzymatic (such as superoxide dismutase, catalase, glutathione reductase, and various peroxidases) and non-enzymatic (such as vitamins C and E, carotenoids, flavonoids, and other phenolic compounds) antioxidants, as recorded in cherry tomatoes [81]. Pea seedlings subjected to salt stress have significantly lower catalase activity than controls [82]. Reduced protein turnover may explain the decline in CAT activity in stressful settings in pea and rye [82,83].

As previously mentioned, GSH plays a significant role in plant metabolism and in the plant’s ability to withstand oxidative stress [84,85]. To prevent enzyme inactivation from thiol group oxidation, GSH can react directly with free radicals, as found in rice [86]. Some species are able to increase the production of antioxidant molecules and enzymes in response to salinity, as reported in *Kandelia candel* [87], and this is linked to their salt tolerance. Salt causes lipid peroxidation of plant cell membranes, yet increased antioxidant enzyme activity helps plants rid themselves of harmful levels of ROS, as reported in wheat and common beans [69,88].

Chitosan can mitigate abiotic stress and help plants avoid oxidative stress by increasing enzyme activity and decreasing lipid peroxidation. This is because of chitosan’s structural makeup and the fact that it can act as a buffer for plants experiencing salt stress, as reported in sweet pepper plants [89]. For instance, tomatoes treated with nanoparticles have been shown to have higher antioxidant levels than those obtained from untreated plants [90,91]. Salinity negatively affected the antioxidant enzymes APX and GR, whereas CsNPs enhanced those antioxidant activities, as reported in *Catharanthus roseus* [41]. In addition, the role of CsNPs in alleviating the harmful effects of salt stress in chilli pepper was revealed by an increase in SOD and CAT activities after the application of the nanoparticles [52].

## 4. Materials and Methods

### 4.1. Plant Material and Treatments

The experiment was carried out in the Botanical Garden at Mansoura University, Faculty of Science in Mansoura, Egypt, to investigate the effects of exogenous application of chitosan (Cs; bulk material) or chitosan nanoparticles (CsNPs) at 0.05% on yield parameters, different pigment fractions, carbohydrates content, ions content, proline, hydrogen peroxide, lipid peroxidation, electrolyte leakage content, and antioxidant system of saline-stressed (i.e., 25, 50, 100, and 200 mM NaCl) common bean plants grown in a clay–sandy soil from November 2021 to February 2022. The first sowing day was 22 November 2021. In the current experiment, the exogenous application of Cs and CsNPs was conducted prior to the salt stress treatments.

A uniformly sized lot of seeds of a pure cultivar from common bean (*Phaseolus vulgaris* L.; cv. Nebraska) was supplied by the Agriculture Research Center, Ministry of Agriculture, Mansoura, Dakahlia Governorate, Mansoura, Egypt. The Nebraska cultivar was chosen for this study as it is widely cultivated in the Mansoura region of Egypt. The seeds were sown in a clay–sandy soil mixture (2:1, *v*/*v*) in pots (30 cm × 28 cm × 26 cm). Each pot contained 10 kg of homogenous soil, in which 10 seeds were planted. Superphosphate was added to the soil prior to sowing at a rate of 14 g per pot.

Thinning was conducted after 10 days of germination, and seven seedlings per pot were kept for experimentation. Thereafter, 75 pots were used for all treatments, which were divided into fifteen groups, each of which contained five pots. One of these groups was left without treatment to serve as water control, and the other fourteen groups were separately treated with either only salinity or salinity combined with exogenous application of chitosan or chitosan nanoparticles. Thus, a penta-replicated, completely randomized design of 15 treatments was represented. The foliar application was applied before salinity stress two times, at 16 and 21 days after sowing. Salinity stress was applied with irrigation water at 0 (C), 25 (S1), 50 (S2), 100 (S3), and 200 (S4) mM NaCl four times at 23, 26, 29, and 32 days after sowing. These concentrations of NaCl refer to the concentration of NaCl in the supplied water. The volume of applied saline solution to each pot was 1500 mL, while the control group was supplied with 1500 mL water.

The surfaces of the pots were covered by a plastic cover during the application of chitosan or chitosan nanoparticles to avoid their entry into the soil network. All pots were irrigated with tap water every three days to maintain the soil water at field capacity throughout the entire period of experimentation. As suggested by preliminary experiments, the concentration of both the bulk chitosan and the chitosan nanoparticles was 0.05%.

The samples corresponding to the stages of flowering and yield were collected 44 and 93 days, respectively, following the date of planting. The samples were collected to include plants from each treatment group in five pots. The allocated samples were used to determine the yield characteristics. Additionally, samples collected during the flowering stage were used to analyze and measure various components, including photosynthetic pigments, carbohydrates, proline, mineral elements, lipid peroxidation, hydrogen peroxide, antioxidant compounds (total phenolics, AsA, and reduced glutathione (GSH)), and antioxidant enzymes (SOD, CAT, APX, GR, POX, and polyphenol oxidase (PPO)). It is important to mention that all measurements were conducted in three replicates, and the mean values are displayed in the corresponding tables and figures.

### 4.2. Preparation and Characterization of Chitosan Nanoparticles

Chitosan with a molecular weight (MW) of 1526.46 g mol^−^^1^ and a deacetylation degree (DD) of 85% was used to prepare chitosan nanoparticles using the methacrylic acid method [44,45,92]. The average size and zeta potential of the obtained chitosan nanoparticles were determined by measuring zeta size using Zetasizer NanoZS (Malvern Instruments, EM unit, Mansoura University, Mansoura, Egypt). On a carbon-coated grid, one drop of the prepared chitosan nanoparticles was spread, and then the grid was dried at room temperature and examined using a JEOL 1010 transmission electron microscope at 80 kV (JEOL, EM unit, Mansoura University, Mansoura, Egypt). The FTIR spectrum of Cs nanoparticles was taken on a Fourier transform spectrometer (NICOLET IS10 FTIR instrument, IR unit, Faculty of Science, Mansoura University, Mansoura, Egypt) in the range from 4000 to 400 cm^−1^. The CsNPs were lyophilized by freeze-drying after being frozen in liquid nitrogen. Pellets were made using these powdered samples and KBr [44].

#### 4.2.1. Morphology and Size of Chitosan Nanoparticles

The results herein indicated that during the synthesis of chitosan nanoparticles, the chitosan solution in methacrylic acid (MAA) changed from a clear to an opalescent suspension. This transformation is visual evidence of the polymerization of chitosan with MAA. The nanoparticles exhibit a very compact structure and have a spherical shape. The chitosan nanoparticles obtained had a mean diameter of 40 ± 2 nm (Supplementary Figure S1).

#### 4.2.2. Zeta Potential

To confirm the stability of the prepared chitosan nanoparticles, the zeta potential (ZP ζ) for CsNPs was measured at pH 4.0. The obtained chitosan nanoparticles showed an average zeta potential of +30.10 mV.

#### 4.2.3. Fourier Transform InfraRed (FTIR) Analysis for CsNPs

FTIR analysis was performed to study spectral changes in position or shape that occurred in the distribution of frequencies of CsNPs, which was used to identify the presence of functional groups. It can be observed that the presence of two new bands at 1646 and 1546 cm^−1^ assigned to COO− and NH_3_^+^ groups, respectively, indicates ionic interaction between MAA and Cs associated with the formation of nanoparticles. The bands at 1741 and 1835 cm^−1^ (C=O) confirm the presence of poly-MAA in the nanoparticle composition (Supplementary Figure S2).

### 4.3. Measurements

#### 4.3.1. Growth and Yield Traits

Growth and yield traits were measured in terms of shoot length, plant height, pod length, pod weight, crop yield/plant, number of pods/plant, number of seeds/pod, seed weight/pod, total seed yield/plant, straw yield, harvest index, crop index, and mobilization index. The harvest index was calculated as the ratio of economic yield (seed yield) to straw yield [93], while the crop index was calculated as the ratio of seed yield to biological yield (seed yield + straw yield). Also, the mobilization index was calculated as the ratio of crop index to straw yield [94].

#### 4.3.2. Photosynthetic Pigment Traits

Photosynthetic pigments (Chl a, Chl b, and carotenoids) were analyzed using the spectrophotometric method [95].

#### 4.3.3. Carbohydrate Fraction Analysis

Using the anthrone method, total carbohydrates were analyzed as described by Hedge and Hofreiter [96]. The content of total soluble sugars (TSS) was also analyzed, as reported by Yemm and Willis [97]. The difference between total carbohydrates and total soluble sugars was used to calculate the starch content.

#### 4.3.4. Na^+^ and K^+^ Contents

Plant tissue of a known dry weight was powdered and digested in concentrated HNO_3,_ and the extract was made up to a known volume with deionized water [98]. By using a flame photometer (PFP7, Jenway, Staffordshire, UK), the amounts of Na^+^ and K^+^ ions were determined. The data were calculated as mg g^−^^1^ dry weight.

#### 4.3.5. Proline Analysis

By incubating 0.1 g of dry tissue powder in 10 cm^3^ of water at 90 °C for one hour, plant water extract was formed, then centrifuged, and the pellet was extracted twice [99]. The combined supernatant volume was completed at 10 cm^3^. By combining 1 cm^3^ of plant water extract with 1 cm^3^ of glacial acetic acid and 1 cm^3^ of the ninhydrin reagent, proline was determined. A boiling water bath was used to incubate the mixture for an hour. The absorbance was measured at 510 nm using a Shimadzu spectrophotometer model UV-160A (Markham, ON, Canada) after cooling.

#### 4.3.6. Hydrogen Peroxide Content

The content of H_2_O_2_ was measured as follows: Common bean leaves weighing about 0.2 g were ground in 5 cm^3^ of TCA. The mixture was centrifuged at 12,000 rpm for 15 min at 4 °C. A reaction mixture contained 2 cm^3^ of KI reagent, 0.5 cm^3^ of plant extract, and 0.5 cm^3^ of phosphate buffer. The reaction was allowed to settle in the dark for one hour, and the absorbance was measured at 390 nm using a Shimadzu spectrophotometer model UV-160A. Using a standard curve formed with known H_2_O_2_ concentrations, the H_2_O_2_ amount was calculated [100].

#### 4.3.7. Lipid Peroxidation

By measuring the amount of the byproduct of unsaturated fatty acid peroxidation, malondialdehyde (MDA), lipid peroxidation was estimated [101]. An aliquot of 5 cm^3^ of 0.1% TCA was used to macerate 1 g of fresh plant material, followed by centrifuging the homogenate at 10,000× *g* for 5 min. For every 1 cm^3^ of the supernatant, 4 cm^3^ of 20% TCA containing 0.5% thiobarbituric acid (TBA) was added. The mixture was quickly cooled in an ice bath after being incubated at 95 °C for 30 min, followed by centrifuging the resulting mixture at 10,000× *g* for 15 min. The absorbance of the supernatant was measured at 532 nm. Measurements were corrected for non-specific turbidity by subtracting the absorbance at 600 nm (A_532_–A_600_). Using an extinction coefficient of 155 mM cm^−^^1^, the amount of MDA was calculated and expressed as µmol MDA g^−^^1^ fresh weight.

#### 4.3.8. Electrolyte Leakage; El of Plant Tissues

Freshly harvested common bean leaves were cut into thin discs. The discs were then placed in a test tube and rinsed with 20 cm^3^ of distilled water three times to extract the released electrolytes upon leaf cutting. After that, 30 cm^3^ of distilled water was added to the tubes and left in the dark at room temperature for one day [102]. Electrical conductivity (EC_1_) was measured by an EC meter (HANNA Instrument, HI 99301, Bedfordshire, UK) once the incubation period had ended. Then the tubes were heated in a boiling water bath at 95 °C for 20 min, after which they were cooled to room temperature. A final electrical conductivity (EC_2_) measurement was made. The following formula was used to determine the electrolyte leakage (El): El = (EC_1_/EC_2_) × 100.

#### 4.3.9. Antioxidant Activity

##### Non-Enzymatic Antioxidant

- Total Phenolic Content

The total phenolic content was estimated as described by Ainsworth and Gillespie [103]. A known dry weight of common bean leaves was ground with 5 cm^3^ of methanol in an ice-cold mortar and pestle, and the mixture was incubated for 48 h in the dark at room temperature. The mixture was then centrifuged at room temperature for five minutes at a speed of 13,000 rpm. To 0.1 cm^3^ of the extract, 0.5 cm^3^ of the F–C reagent and 0.8 cm^3^ of the Na_2_CO_3_ solution were added. The tubes were then kept at room temperature for two hours. At 765 nm, the absorbance was measured with reference to a methanol blank. The phenolics concentration was expressed as mg gallic acid equivalent/g DW of the sample.

- Ascorbic acid (AsA)

The AsA content was calculated [104]. About 0.2 g of fresh common bean leaves or seeds were homogenized in 10 cm^3^ of ice-cold TCA. After that, the mixture was centrifuged for 20 min at 4 °C and 3500 rpm. To form the bis-2,4-dinitrophenylhydrazone derivative, a mixture of 0.5 cm^3^ supernatant and 0.1 cm^3^ DTC was incubated for three hours at 37 °C. To convert this into the rearranged product, which was measured spectrophotometrically, 0.75 cm^3^ of ice-cold 65% H_2_SO_4_ was added and mixed well, and an additional 30 min at room temperature were given for the solutions to stand. At 520 nm, absorbances were measured. The values were expressed as mg of AsA/g fresh weight.

- Reduced glutathione (GSH)

The amount of reduced glutathione was calculated [105]. About 0.2 g of fresh common bean leaves were homogenized in 5 cm^3^ of 50 mM phosphate buffer (pH 7.5 in EDTA solution). The homogenate was then centrifuged for 20 min at 14,000 rpm. After mixing 0.5 cm^3^ of the supernatant with 0.5 cm^3^ of TCA, the mixture was allowed to stand at room temperature for 5 min, and 0.1 cm^3^ of 0.1 mM DTNB and 1 cm^3^ of 0.1 M phosphate buffer were added to the reaction mixture. After 5 min, the 405 nm wavelength was used to measure the absorbance of the yellow color created by the reaction of GSH with DTNB. The values were expressed as mmoles GSH/g leaf.

##### Activity of Antioxidant Enzymes

Enzyme extraction: About 200 mg of fresh common bean leaves were extracted in 5 cm^3^ chilled phosphate buffer. The 0.1 M phosphate buffer at pH 7.8 was used for APX and SOD. The 0.1 M phosphate buffer was used at a pH of 6.8 for CAT, GR, POX, and PPO. The homogenate was filtered through cheesecloth before being centrifuged at 10,000 rpm for 20 min in a chilled centrifuge. The supernatant was the enzyme extract. Every procedure was carried out at 4 °C [106].

- Catalase Activity (CAT, EC 1.11.1.6).

To begin the reaction, 1 cm^3^ of potassium phosphate buffer (0.1 M, pH 7.5) and 0.2 cm^3^ of 30% H_2_O_2_ (prepared immediately before use) were added to 1.5 cm^3^ of enzyme extract, and the activity was measured at 240 nm for one minute by observing H_2_O_2_ removal [107]. One unit of catalase will decompose 1.0 µmole of H_2_O_2_ per minute.

- Peroxidase Activity (POX, EC 1.11.1.7).

POX activity was estimated at 420 nm as an increase in absorption caused by purpurogallin formation [108]. The reaction mixture included 0.1 cm^3^ enzyme extract, 3 cm^3^ of pyrogallol phosphate buffer (0.05 M pyrogallol in 0.1 M phosphate buffer, pH 6), and 0.5 cm^3^ of 1% H_2_O_2_. One unit of enzyme is defined as one unit per gram of fresh weight per min. One unit of peroxidase will form 1.0 mg of purpurogallin from pyrogallol in 20 s.

- Polyphenol Oxidase Activity (PPO, EC 1.10.3.1).

PPO activity was measured at 420 nm as an increase in absorbance by the formation of purpurogallin [108]. The reaction mixture consisted of 1 cm^3^ of 0.1 M pyrogallol, 2 cm^3^ of 0.02 M phosphate buffer (pH 7), and 1 cm^3^ of the enzyme extract. The reaction mixture was kept at 25 °C for one minute, and the reaction was stopped by adding 1 mL of 2.5 N H_2_SO_4_. One enzyme unit is defined as a unit per gram of fresh weight per min. An increase in absorbance of 0.001 min^−1^ was taken as one unit of enzyme activity.

- Ascorbate Peroxidase Activity (APX, EC 1.11.1.11).

By measuring the 290 nm decrease in absorbance caused by ascorbate oxidation, the activity of ascorbate peroxidase was determined [109]. The reaction mixture contained 0.13 cm^3^ of 2 mM H_2_O_2,_ 0.83 cm^3^ of 0.5 mM AsA in phosphate buffer (pH 7), and 0.04 cm^3^ of enzyme extract at a final volume of 1 cm^3^ at 25 °C. One unit of enzyme activity was calculated as the amount of enzyme required to oxidize 1.0 μmol of ascorbate/min/g FW.

- Superoxide Dismutase Activity (SOD, EC 1.15.1.1).

SOD activity was estimated in the current study [110]. One SOD activity enzyme unit is defined as the amount of enzyme needed for 50% inhibition of the reduction in nitro-bluetetrazolium (NBT) at 560 nm. The reaction mixture included 1 cm^3^ of 1 mM NBT and 1 cm^3^ of 1 mM NADH, 1 cm^3^ of working buffer (50 mM phosphate buffer; pH 8.5), 0.1 cm^3^ of enzyme extract, mixed well, and 0.1 cm^3^ of 0.1 mM phenazine methosulphate (PMS). The ability of the enzyme extract to prevent a photochemical reduction in NBT was used to measure SOD activity.

- Glutathione Reductase Activity (GR, EC 1.8.1.7).

GR activity was measured as a 340 nm decrease in absorbance caused by GSSG reduction in the presence of NADPH, which is oxidized to NADP^+^ [111]. The reaction mixture contained 0.1 cm^3^ of 50 mM GSSG, 0.1 cm^3^ of 2 mM NADPH, 1 cm^3^ of 100 mM potassium phosphate-EDTA buffer (pH 7.5), and 0.05 cm^3^ of enzyme extract at a final volume of 1.25 cm^3^. A unit of GR activity was defined as the amount of enzyme that catalyzes the reduction of 1 µmol of oxidized glutathione (GSSG) per minute.

### 4.4. Statistical Analysis

The data were statistically processed by two-way analysis of variance (ANOVA) with the post hoc Duncan test, using SPSS software version 22. The significance of differences between the means was tested at *p* ≤ 0.05.

## 5. Conclusions

In conclusion, our study concluded that salinity stress significantly and negatively affected all growth, physiological, biochemical, and yield traits. However, plants treated with exogenous application of Cs or CsNPs at 0.05% before the application of salt stress treatments showed an improvement in all growth and developmental traits. It was apparent that CsNPs were more beneficial in mitigating salt stress compared to the application of Cs, which suggests the use of CsNPs in salinity stress tolerance with other crops. However, further research is needed to determine the long-term impact of CsNPs on crop productivity for crop species and the limits of their application.

## Figures and Tables

**Table 1:** Effects of exogenous application with chitosan or chitosan nanoparticles on growth and pod traits of common bean plants treated with different levels of salt stress at maturity (i.e., at 93 days from planting).

Treatments		Plant Height (cm)	Shoot Length (cm)	Pod Length (cm)	Plant Water Content (%)	Number of Pods/Plant	Pod Weight (g/pod)	Number of Seeds/Pod	Seeds Weight (g/pod)
Control	No Cs	77.20 ^d^ ± 3.20	69.80 ^e^ ± 2.25	11.00 ^c^ ± 0.23	80.27 ^f^ ± 1.22	5.1 ^a^ ± 0.13	3.26 ^c^ ± 0.23	3.2 ^a^ ± 0.11	0.29 ^f^ ± 0.03
	Cs	85.00 ^c^ ± 2.10	79.00 ^c^ ± 1.23	11.60 ^b^ ± 0.33	78.58 ^l^ ± 1.02	6.2 ^a^ ± 0.12	3.52 ^b^ ± 0.12	4.1 ^a^ ± 0.21	0.41 ^b^ ± 0.04
	CsNPs	96.00 ^a^ ± 3.23	88.50 ^a^ ± 1.20	12.00 ^a^ ± 0.43	79.88 ^g^ ± 1.33	6.2 ^a^ ± 0.22	3.59 ^a^ ± 0.16	5.0 ^a^ ± 0.13	0.48 ^a^ ± 0.05
S1	No Cs	71.60 ^e^ ± 2.25	63.50 ^fg^ ± 2.20	10.25 ^d^ ± 0.53	79.23 ^i^ ± 2.22	5.4 ^a^ ± 0.13	3.18 ^d^ ± 0.23	3.2 ^a^ ± 0.33	0.26 ^g^ ± 0.06
	Cs	78.00 ^d^ ± 3.44	66.40 ^f^ ± 4.20	10.80 ^c^ ± 0.27	79.25 ^i^ ± 0.92	5.3 ^a^ ± 0.24	3.46 ^b^ ± 0.16	4.1 ^a^ ± 0.23	0.35 ^d^ ± 0.05
	CsNPs	91.00 ^b^ ± 3.23	85.25 ^b^ ± 3.26	11.50 ^b^ ± 0.43	78.63 ^k^ ± 0.98	6.1 ^a^ ± 0.33	3.51 ^b^ ± 0.27	4.1 ^a^ ± 0.25	0.46 ^a^ ± 0.04
S2	No Cs	64.10 ^g^ ± 2.12	56.00 ^h^ ± 1.60	9.75 ^e^ ± 0.63	79.46 ^h^ ± 1.52	5.2 ^a^ ± 0.13	2.85 ^f^ ± 0.13	3.1 ^a^ ± 0.26	0.21 ^h^ ± 0.03
	Cs	72.50 ^e^ ± 1.45	63.00 ^g^ ± 3.77	10.30 ^d^ ± 0.33	78.40 ^m^ ± 1.44	5.2 ^a^ ± 0.23	3.06 ^e^ ± 0.23	4.1 ^a^ ± 0.21	0.27 ^fg^ ± 0.04
	CsNPs	89.00 ^b^ ± 2.66	81.25 ^c^ ± 3.28	10.90 ^c^ ± 0.73	79.07 ^j^ ± 1.32	6.1 ^a^ ± 0.25	3.13 ^d^ ± 0.43	4.1 ^a^ ± 0.13	0.38 ^c^ ± 0.05
S3	No Cs	57.50 ^h^ ± 5.10	49.25 ^i^ ± 4.20	8.25 ^h^ ± 0.23	82.79 ^b^ ± 0.92	3.0 ^a^ ± 0.13	2.42 ^h^ ± 0.23	2.0 ^a^ ± 0.14	0.16 ^i^ ± 0.03
	Cs	67.25 ^f^ ± 4.20	61.25 ^g^ ± 2.25	9.00 ^f^ ± 0.53	82.13 ^c^ ± 0.82	3.0 ^a^ ± 0.12	2.66 ^g^ ± 0.13	3.2 ^a^ ± 0.13	0.19 ^h^ ± 0.02
	CsNPs	79.00 ^d^ ± 1.20	74.75 ^d^ ± 1.70	9.50 ^e^ ± 0.65	81.37 ^e^ ± 1.20	4.1 ^a^ ± 0.24	2.89 ^f^ ± 0.25	4.0 ^a^ ± 0.12	0.32 ^e^ ± 0.01
S4	No Cs	52.40 ^i^ ± 3.26	46.20 ^j^ ± 3.67	6.75 ^i^ ± 0.67	81.60 ^d^ ± 1.18	3.0 ^a^ ± 0.25	2.11 ^k^ ± 0.33	2.2 ^a^ ± 0.15	0.13 ^j^ ± 0.02
	Cs	62.00 ^g^ ± 2.66	56.50 ^h^ ± 2.20	8.10 ^h^ ± 0.43	81.63 ^d^ ± 1.09	3.1 ^a^ ± 0.33	2.20 ^j^ ± 0.13	3.2 ^a^ ± 0.16	0.14 ^ij^ ± 0.03
	CsNPs	76.80 ^d^ ± 1.70	70.25 ^e^ ± 1.29	8.70 ^g^ ± 0.53	83.37 ^a^ ± 1.11	4.1 ^a^ ± 0.43	2.31 ^i^ ± 0.11	3.2 ^a^ ± 0.25	0.25 ^g^ ± 0.04

S1, S2, S3, and S4 are 25, 50, 100, and 200 mM NaCl, respectively. Chitosan (Cs) and chitosan nanoparticles (CsNPs) were applied at 0.05%. Means (of three replicates ± standard error) in each column followed by a similar letter are not significantly different at *p* ≤ 0.05 using the Duncan test.

**Table 2:** Effects of exogenous application with chitosan or chitosan nanoparticles on yield traits of common bean plants treated with different levels of salt stress at maturity (i.e., at 93 days from planting).

Treatments		Crop Yield (g/Plant)	Seed Yield (g/Plant)	Straw Yield (g/plant)	Harvest Index	Crop Index	Mobilization Index
Control	No Cs	5.44 ^e^ ± 0.23	3.53 ^cd^ ± 0.13	2.52 ^d^ ± 0.26	1.40 ^f^ ± 0.13	0.58 ^e^ ± 0.03	2.16 ^e^ ± 0.13
	Cs	6.27 ^c^ ± 0.13	4.02 ^b^ ± 0.21	2.81 ^cd^ ± 0.33	1.43 ^e^ ± 0.11	0.59 ^d^ ± 0.02	2.23 ^b^ ± 0.12
	CsNPs	7.54 ^a^ ± 0.23	5.34 ^a^ ± 0.14	3.48 ^a^ ± 0.13	1.53 ^c^ ± 0.10	0.61 ^b^ ± 0.05	2.17 ^d^ ± 0.15
S1	No Cs	4.75 ^g^ ± 0.25	3.22 ^de^ ± 0.12	2.21 ^e^ ± 0.21	1.46 ^d^ ± 0.20	0.59 ^d^ ± 0.06	2.15 ^f^ ± 0.17
	Cs	5.76 ^d^ ± 0.13	3.61 ^c^ ± 0.14	2.73 ^cd^ ± 0.25	1.32 ^g^ ± 0.13	0.57 ^f^ ± 0.07	2.11 ^h^ ± 0.23
	CsNPs	7.22 ^b^ ± 0.27	5.10 ^a^ ± 0.21	3.27 ^ab^ ± 0.28	1.56 ^b^ ± 0.11	0.61 ^b^ ± 0.02	2.21 ^c^ ± 0.22
S2	No Cs	4.21 ^h^ ± 0.13	2.99 ^ef^ ± 0.14	1.96 ^ef^ ± 0.13	1.53 ^c^ ± 0.12	0.60 ^c^ ± 0.03	2.15 ^f^ ± 0.16
	Cs	5.08 ^f^ ± 0.25	3.25 ^de^ ± 0.21	2.55 ^d^ ± 0.22	1.27 ^h^ ± 0.14	0.56 ^g^ ± 0.04	1.99 ^l^ ± 0.33
	CsNPs	6.53 ^c^ ± 0.13	4.21 ^b^ ± 0.15	3.02 ^bc^ ± 0.13	1.39 ^f^ ± 0.11	0.58 ^e^ ± 0.03	2.16 ^e^ ± 0.23
S3	No Cs	3.33 ^jk^ ± 0.33	2.48 ^h^ ± 0.26	1.57 ^g^ ± 0.21	1.58 ^b^ ± 0.11	0.61 ^b^ ± 0.05	2.12 ^g^ ± 0.33
	Cs	3.65 ^i^ ± 0.13	2.85 ^fg^ ± 0.17	1.82 ^fg^ ± 0.23	1.57 ^b^ ± 0.13	0.61 ^b^ ± 0.02	2.01 ^k^ ± 0.13
	CsNPs	4.11 ^h^ ± 0.23	3.05 ^ef^ ± 0.28	2.16 ^e^ ± 0.16	1.41 ^ef^ ± 0.11	0.59 ^d^ ± 0.05	1.90 ^m^ ± 0.23
S4	No Cs	3.05 ^k^ ± 0.24	2.07 ^i^ ± 0.33	1.23 ^h^ ± 0.27	1.68 ^a^ ± 0.10	0.63 ^a^ ± 0.05	2.48 ^a^ ± 0.23
	Cs	3.24 ^k^ ± 0.13	2.39 ^h^ ± 0.13	1.58 ^g^ ± 0.21	1.51 ^c^ ± 0.14	0.60 ^c^ ± 0.03	2.05 ^i^ ± 0.33
	CsNPs	3.57 ^ij^ ± 0.22	2.58 ^gh^ ± 0.25	1.75 ^fg^ ± 0.13	1.47 ^d^ ± 0.15	0.60 ^c^ ± 0.02	2.04 ^j^ ± 0.33

For definitions of treatments’ abbreviations, see the bottom of Table 1. Means (of three replicates ± standard error) in each column followed by a similar letter are not significantly different at *p* ≤ 0.05 using the Duncan test.

**Table 3:** Summary of the two-way ANOVA showing the effect of the main factors: chitosan spray and salinity and their interaction on yield traits of common bean plants at maturity (i.e., at 93 days from planting).

Variable and Source of Variation	df	F	*p*	Variable and Source of Variation	df	F	*p*
**Plant height**				**Crop yield**			
Chitosan	2	3842.48	***	Chitosan	2	1174.09	***
Salinity	4	1553.80	***	Salinity	4	1942.04	***
Chitosan × Salinity	8	15.64	***	Chitosan × Salinity	8	73.49	***
**Shoot length**				**Seed yield**			
Chitosan	2	2356.03	***	Chitosan	2	761.92	***
Salinity	4	733.40	***	Salinity	4	794.15	***
Chitosan × Salinity	8	20.30	***	Chitosan × Salinity	8	54.52	***
**Pod length**				**Straw yield**			
Chitosan	2	102.96	***	Chitosan	2	302.20	***
Salinity	4	309.90	***	Salinity	4	374.26	***
Chitosan × Salinity	8	2.02	ns	Chitosan × Salinity	8	7.32	***
**Plant water content**				**Harvest index**			
Chitosan	2	4444.85	***	Chitosan	2	10.81	***
Salinity	4	60,066.80	***	Salinity	4	8.34	***
Chitosan × Salinity	8	4000.85	***	Chitosan × Salinity	8	7.59	***
**Number of pods/plant**				**Crop index**			
Chitosan	2	4.20	*	Chitosan	2	10.40	***
Salinity	4	12.20	***	Salinity	4	12.30	***
Chitosan × Salinity	8	0.20	ns	Chitosan × Salinity	8	8.40	***
**Pod weight**				**Mobilization index**			
Chitosan	2	44.92	***	Chitosan	2	154.66	***
Salinity	4	271.85	***	Salinity	4	98.94	***
Chitosan × Salinity	8	1.07	ns	Chitosan × Salinity	8	82.66	***
**Number of seeds/pod**							
Chitosan	2	7.80	**				
Salinity	4	2.70	*				
Chitosan × Salinity	8	0.30	ns				
**Seeds weight**							
Chitosan	2	341.22	***				
Salinity	4	221.19	***				
Chitosan × Salinity	8	5.38	***				

* *p* ≤ 0.05, ** *p* ≤ 0.01, *** *p* ≤ 0.001, ns = non-significant.

**Table 4:** Effects of exogenous application of chitosan or chitosan nanoparticles on different photosynthetic pigment fractions, total soluble sugars (TSS), starch, and total carbohydrates of common bean plants treated with different levels of salt stress at the flowering stage (i.e., 44 days from planting).

Treatments		Chlorophyll a	Chlorophyll b	Carotenoids	Total Pigments	TSS	Starch	Total Carbohydrates
		(mg/g FW)				(mg Glucose Equivalent/g DW)		
Control	No Cs	1.92 ^g^ ± 0.13	0.60 ^bc^ ± 0.03	1.03 ^abc^ ± 0.13	3.55 ^cde^ ± 0.23	56.12 ^j^ ± 3.23	355.30 ^c^ ± 13.23	411.42 ^cd^ ± 43.33
	Cs	2.28 ^b^ ± 0.17	0.62 ^b^ ± 0.02	1.04 ^ab^ ± 0.14	3.94 ^b^ ± 0.25	58.82 ^i^ ± 4.25	401.29 ^b^ ± 15.26	460.11 ^b^ ± 44.14
	CsNPs	2.49 ^a^ ± 0.12	0.67 ^a^ ± 0.03	1.05 ^a^ ± 0.17	4.21 ^a^ ± 0.27	62.80 ^h^ ± 3.45	441.08 ^a^ ± 26.33	503.88 ^a^ ± 35.34
S1	No Cs	1.88 ^h^ ± 0.13	0.55 ^ef^ ± 0.04	1.01 ^c^ ± 0.16	3.44 ^de^ ± 0.28	69.50 ^g^ ± 3.44	316.00 ^e^ ± 23.27	385.50 ^e^ ± 43.33
	Cs	2.19 ^d^ ± 0.12	0.58 ^cd^ ± 0.05	1.02 ^bc^ ± 0.17	3.79 ^bc^ ± 0.33	73.93 ^f^ ± 4.26	359.16 ^c^ ± 27.29	433.09 ^c^ ± 56.25
	CsNPs	2.25 ^bc^ ± 0.13	0.59 ^c^ ± 0.06	1.04 ^ab^ ± 0.13	3.88 ^b^ ± 0.36	76.58 ^e^ ± 4.26	403.01 ^b^ ± 33.43	479.59 ^b^ ± 33.23
S2	No Cs	1.83 ^i^ ± 0.15	0.53 ^fg^ ± 0.03	0.97 ^d^ ± 0.14	3.36 ^ef^ ± 0.24	78.89 ^d^ ± 3.24	273.27 ^f^ ± 43.23	352.16 ^fg^ ± 13.23
	Cs	2.14 ^e^ ± 0.16	0.56 ^de^ ± 0.04	1.01 ^c^ ± 0.15	3.71 ^bcd^ ± 0.43	80.72 ^d^ ± 3.33	310.26 ^e^ ± 33.25	390.98 ^de^ ± 33.43
	CsNPs	2.23 ^c^ ± 0.17	0.58 ^cd^ ± 0.06	1.02 ^bc^ ± 0.12	3.86 ^bc^ ± 0.26	85.47 ^c^ ± 3.43	334.61 ^d^ ± 43.20	420.08 ^c^ ± 53.23
S3	No Cs	1.78 ^j^ ± 0.13	0.47 ^h^ ± 0.04	0.87 ^f^ ± 0.13	3.12 ^fg^ ± 0.27	85.97 ^c^ ± 3.53	222.27 ^h^ ± 45.21	308.24 ^h^ ± 53.23
	Cs	2.06 ^f^ ± 0.15	0.51 ^g^ ± 0.03	0.92 ^e^ ± 0.15	3.49 ^de^ ± 0.43	87.34 ^bc^ ± 3.33	268.05 ^f^ ± 34.43	355.39 ^f^ ± 53.26
	CsNPs	2.16 ^de^ ± 0.12	0.54 ^ef^ ± 0.02	0.95 ^d^ ± 0.13	3.68 ^bcd^ ± 0.33	89.39 ^ab^ ± 4.23	272.10 ^f^ ± 45.23	361.49 ^f^ ± 63.23
S4	No Cs	1.67 ^l^ ± 0.11	0.35 ^j^ ± 0.06	0.83 ^g^ ± 0.17	2.88 ^g^ ± 0.43	89.07 ^ab^ ± 5.27	176.95 ^i^ ± 55.55	266.02 ^i^ ± 65.23
	Cs	1.73 ^k^ ± 0.12	0.38 ^i^ ± 0.05	0.87 ^f^ ± 0.18	3.01 ^g^ ± 0.23	90.53 ^a^ ± 6.39	220.52 ^h^ ± 44.27	311.05 ^h^ ± 67.23
	CsNPs	1.82 ^i^ ± 0.14	0.40 ^i^ ± 0.08	0.91 ^e^ ± 0.12	3.16 ^fg^ ± 0.13	91.12 ^a^ ± 5.25	239.50 ^g^ ± 56.23	330.62 ^gh^ ± 77.23

For definitions of treatments’ abbreviations, see the bottom of Table 1. Means (of three replicates ± standard error) in each column followed by a similar letter are not significantly different at *p* ≤ 0.05 using the Duncan test.

**Table 5:** Summary of the two-way ANOVA showing the effect of the main factors: chitosan spray and salinity and their interaction on different photosynthetic pigment fractions, total soluble sugars (TSS), starch, and total carbohydrates of common bean plants at the flowering stage (i.e., 44 days from planting).

Variable and Source of Variation	df	F	*p*	Variable and Source of Variation	df	F	*p*
**Chlorophyll a**				**TSS**			
Chitosan	2	1629.94	***	Chitosan	2	436.89	***
Salinity	4	894.94	***	Salinity	4	6140.95	***
Chitosan × Salinity	8	58.10	***	Chitosan × Salinity	8	23.99	***
**Chlorophyll b**				**Starch**			
Chitosan	2	117.80	***	Chitosan	2	19,333.83	***
Salinity	4	824.30	***	Salinity	4	54,486.97	***
Chitosan × Salinity	8	2.30	*	Chitosan × Salinity	8	285.36	***
**Carotenoids**				**Total Carbohydrates**			
Chitosan	2	102.20	***	Chitosan	2	21,131.50	***
Salinity	4	491.70	***	Salinity	4	36,793.48	***
Chitosan × Salinity	8	6.45	***	Chitosan × Salinity	8	312.38	***
**Total pigments**							
Chitosan	2	9204.20	***				
Salinity	4	10,190.80	***				
Chitosan × Salinity	8	179.20	***				

* *p* ≤ 0.05, *** *p* ≤ 0.001.

**Table 6:** Effects of exogenous application of chitosan or chitosan nanoparticles on total phenols, ascorbic acid, reduced glutathione (GSH), catalase (CAT), peroxidase (POX), polyphenol oxidase (PPO), ascorbate peroxidase (APX), superoxide dismutase (SOD), and glutathione reductase (GR) in common bean plants treated with different salt stress levels at flowering stage (i.e., 44 days from planting).

Treatments		Total Phenols (mg/g DW)	Ascorbic Acid (mg/g FW)	GSH (mmol/g FW)	CAT (mmol H_2_O_2_/g FW/min)	POX	PPO	APX (mmol AsA/g FW/min)	SOD	GR
						(U/g FW/min)			(U/g FW/min)	
Control	No Cs	6.95 ^j^ ± 0.13	2.41 ^h^ ± 0.11	1.07 ^l^ ± 0.15	3.18 ^f^ ± 0.18	49.60 ^h^ ± 0.83	20.10 ^k^ ± 0.55	9.79 ^i^ ± 0.33	32.31 ^l^ ± 0.83	4.99 ^o^ ± 0.13
	Cs	7.19 ^ij^ ± 0.17	2.82 ^fg^ ± 0.13	1.66 ^k^ ± 0.13	3.30 ^ef^ ± 0.13	52.00 ^gh^ ± 0.99	24.85 ^j^ ± 0.34	10.07 ^hi^ ± 0.23	37.79 ^i^ ± 0.76	5.65 ^n^ ± 0.14
	CsNPs	8.88 ^fg^ ± 0.16	3.23 ^e^ ± 0.17	1.74 j ± 0.12	3.45 ^e^ ± 0.12	53.30 ^fg^ ± 1.13	25.10 ^j^ ± 0.45	10.39 ^h^ ± 0.32	38.63 ^h^ ± 0.45	6.63 ^m^ ± 0.03
S1	No Cs	7.28 ^ij^ ± 0.14	2.55 ^gh^ ± 0.12	1.89 ^i^ ± 0.11	2.96 ^g^ ± 0.18	50.60 ^h^ ± 0.78	25.35 ^j^ ± 0.43	12.39 ^g^ ± 0.54	34.94 ^k^ ± 0.46	7.23 ^l^ ± 0.21
	Cs	8.57 ^g^ ± 0.23	3.04 ^ef^ ± 0.13	1.93 ^i^ ± 0.13	4.71 ^d^ ± 0.12	55.70 ^ef^ ± 0.63	35.95 ^g^ ± 0.23	13.25 ^f^ ± 0.23	38.90 ^gh^ ± 0.63	9.96 ^k^ ± 0.04
	CsNPs	9.16 ^f^ ± 0.24	3.57 ^cd^ ± 0.15	2.05 ^h^ ± 0.12	4.96 ^c^ ± 0.10	59.60 ^cd^ ± 0.93	39.22 ^f^ ± 0.45	16.39 ^d^ ± 0.44	39.21 ^fg^ ± 0.34	11.12 ^j^ ± 0.15
S2	No Cs	7.59 ^i^ ± 0.17	2.73 ^g^ ± 0.16	2.07 ^h^ ± 0.10	2.57 ^h^ ± 0.23	53.70 fg ± 0.63	28.75 ^i^ ± 0.23	13.39 ^f^ ± 0.15	37.08 ^j^ ± 0.66	13.36 ^i^ ± 0.23
	Cs	9.31 ^f^ ± 0.18	3.32 ^de^ ± 0.23	2.26 ^g^ ± 0.15	4.71 ^d^ ± 0.11	58.30 ^d^ ± 0.55	41.70 ^e^ ± 0.65	14.04 ^e^ ± 0.25	40.06 ^e^ ± 0.76	17.21 ^h^ ± 0.17
	CsNPs	10.75 ^d^ ± 0.12	3.71 ^c^ ± 0.21	2.65 ^f^ ± 0.12	5.06 ^c^ ± 0.13	61.30 ^c^ ± 1.13	45.10 ^cd^ ± 0.53	18.93 ^c^ ± 0.26	43.19 ^d^ ± 0.33	20.81 ^f^ ± 0.12
S3	No Cs	8.14 ^h^ ± 0.13	3.24 ^e^ ± 0.13	2.67 ^f^ ± 0.13	1.97 ^i^ ± 0.12	57.40 ^de^ ± 0.73	31.25 ^h^ ± 0.52	16.07 ^d^ ± 0.35	39.35 ^f^ ± 0.22	20.43 ^g^ ± 0.19
	Cs	10.03 ^e^ ± 0.22	3.73 ^c^ ± 0.11	3.74 ^d^ ± 0.12	5.79 ^b^ ± 0.10	61.30 ^c^ ± 0.93	43.85 ^d^ ± 0.46	16.32 ^d^ ± 0.37	43.19 ^d^ ± 0.43	27.01 ^d^ ± 0.20
	CsNPs	13.04 ^c^ ± 0.23	4.21 ^b^ ± 0.15	3.96 ^c^ ± 0.10	5.86 ^b^ ± 0.14	71.80 ^a^ ± 0.88	48.20 ^b^ ± 0.54	19.13 ^c^ ± 0.43	46.21 ^c^ ± 0.76	30.21 ^c^ ± 0.23
S4	No Cs	9.16 ^f^ ± 0.15	3.77 ^c^ ± 0.16	3.52 ^e^ ± 0.22	1.54 ^j^ ± 0.21	58.00 ^de^ ± 0.99	35.40 ^g^ ± 0.35	19.00 ^c^ ± 0.23	40.37 ^e^ ± 0.27	26.53 ^e^ ± 0.25
	Cs	13.91 ^b^ ± 0.13	4.12 ^b^ ± 0.17	4.45 ^b^ ± 0.14	5.89 ^b^ ± 0.12	67.70 ^b^ ± 1.33	46.10 ^c^ ± 0.36	19.55 ^b^ ± 0.31	47.92 ^b^ ± 0.65	35.48 ^b^ ± 0.17
	CsNPs	17.73 ^a^ ± 0.11	4.73 ^a^ ± 0.18	4.67 ^a^ ± 0.25	6.13 ^a^ ± 0.11	73.50 ^a^ ± 0.73	50.25 ^a^ ± 0.67	20.17 ^a^ ± 0.53	50.63 ^a^ ± 0.66	40.73 ^a^ ± 0.34

For definitions of treatments’ abbreviations, see the bottom of Table 1. Means (of three replicates ± standard error) in each column followed by a similar letter are not significantly different at *p* ≤ 0.05 using the Duncan test.

**Table 7:** Summary of the two-way ANOVA showing the effect of the main factors: chitosan spray and salinity and their interaction on total phenols, ascorbic acid, reduced glutathione (GSH), catalase (CAT), peroxidase (POX), polyphenol oxidase (PPO), ascorbate peroxidase (APX), superoxide dismutase (SOD), and glutathione reductase (GR) in common bean plants at flowering stage (i.e., 44 days from planting).

Variable and Source of Variation	df	F	*p*	Variable and Source of Variation	df	F	*p*
**Total Phenols**				**PPO**			
Chitosan	2	7626.62	***	Chitosan	2	1814.62	***
Salinity	4	5966.54	***	Salinity	4	1442.85	***
Chitosan × Salinity	8	746.09	***	Chitosan × Salinity	8	45.55	***
**Ascorbic Acid**				**APX**			
Chitosan	2	361.62	***	Chitosan	2	4254.31	***
Salinity	4	296.83	***	Salinity	4	13,778.33	***
Chitosan × Salinity	8	1.18	ns	Chitosan × Salinity	8	470.63	***
**GSH**				**SOD**			
Chitosan	2	386.94	***	Chitosan	2	19,347.31	***
Salinity	4	1832.91	***	Salinity	4	15,780.54	***
Chitosan × Salinity	8	34.41	***	Chitosan × Salinity	8	521.59	***
**CAT**				**GR**			
Chitosan	2	54,128.93	***	Chitosan	2	2744.57	***
Salinity	4	3745.88	***	Salinity	4	16,250.13	***
Chitosan × Salinity	8	4783.31	***	Chitosan × Salinity	8	251.02	***
**POX**							
Chitosan	2	1219.61	***				
Salinity	4	1048.48	***				
Chitosan × Salinity	8	70.94	***				

*** *p* ≤ 0.001, ns = non-significant.

## Data Availability

Data is contained within the article and supplementary materials.

## Supplementary Materials

The following supporting information can be downloaded at: https://www.mdpi.com/article/10.3390/plants13030398/s1, Figure S1: Transmission electron micrograph of chitosan nanoparticles (CsNPs); Figure S2: FT-IR spectrum of pure solid chitosan nanoparticles (CsNPs); Table S1: Effects of exogenous application of chitosan or chitosan nanoparticles on Na^+^, K^+^ contents and Na^+^/K^+^ ratio in shoots and roots of salt stressed common bean plants at flowering stage; Table S2: Effects of exogenous application of chitosan or chitosan nanoparticles on proline, hydrogen peroxide, lipid peroxidation and electrolyte leakage content of salt-stressed common bean plants at flowering stage grown in clay-sandy soil; Table S3: Summary of the Two-way ANOVA showing the effect of the main factors: chitosan spray and salinity and their interaction on Na^+^, K^+^ contents and Na^+^/K^+^ ratio in shoots and roots of salt stressed common bean plants and proline, hydrogen peroxide, lipid peroxidation and electrolyte leakage content at flowering stage.
