# Supplementary material for: Chitosan and Chitosan Nanoparticles Differentially Alleviate Salinity Stress in Phaseolus vulgaris L. Plants
Source: Plants (Basel). 2024 Jan 29;13(3):398. doi: 10.3390/plants13030398 (PMC10857083; doi:10.3390/plants13030398)
Supplement: Supplementary file 1 [file plants-13-00398-s001.zip › plants-2721936-supplementary.pdf]

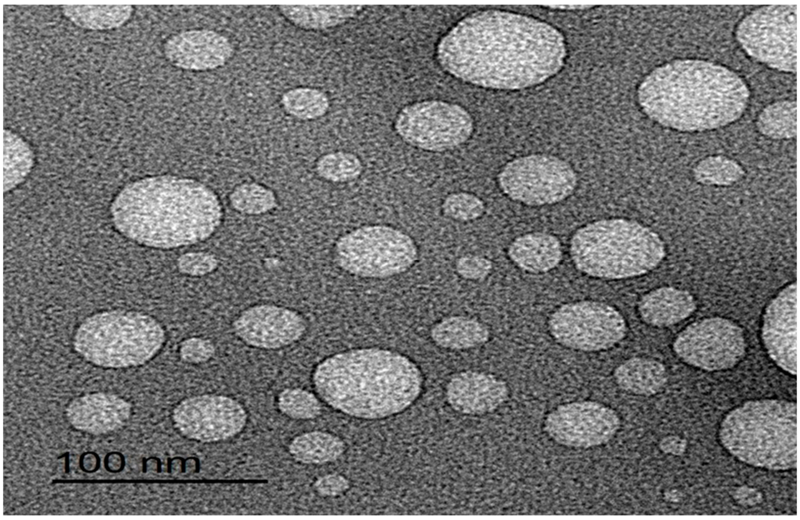

Supplementary Figure S1. Transmission electron micrograph of chitosan nanoparticles (CsNPs).

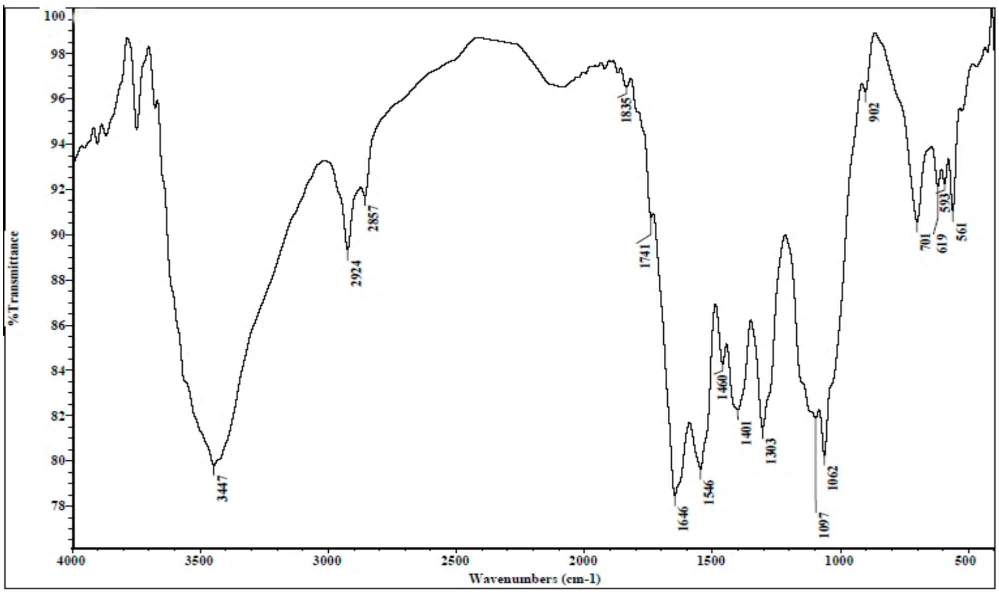

Supplementary Figure S2. FT-IR spectrum of pure solid chitosan nanoparticles (CsNPs).

Supplementary Table S1: Effects of exogenous application of chitosan or chitosan nanoparticles on Na^+, K^+ contents and Na^+/K^+ ratio in shoots and roots of salt stressed common bean plants at flowering stage.

Treatments	Shoot			Root		
	Na^+ (mg/ g DW)	K^+ (mg/ g DW)	Na^+/K^+ ratio	Na^+ (mg/ g DW)	K^+ (mg/ g DW)	Na^+/K^+ ratio
Control	$0.29^{l}\pm0.03$	$1.45^{cd}\pm0.04$	$0.20^{l}\pm0.04$	$0.62^{l}\pm0.04$	$0.58^{cd}\pm0.02$	$1.07^{k}\pm0.02$
S1	$0.33^{j}\pm0.02$	$1.42^{def}\pm0.06$	$0.23^{j}\pm0.03$	$0.71^{j}\pm0.02$	$0.55^{de}\pm0.05$	$1.29^{i}\pm0.03$
S2	$0.48^{g}\pm0.04$	$1.37^{gh}\pm0.05$	$0.35^{g}\pm0.05$	$0.85^{g}\pm0.06$	$0.52^{e}\pm0.04$	$1.63^{g}\pm0.05$
S3	$0.63^{d}\pm0.04$	$1.28^{i}\pm0.04$	$0.49^{d}\pm0.03$	$1.36^{d}\pm0.04$	$0.45^{fg}\pm0.04$	$3.02^{d}\pm0.03$
S4	$0.76^{a}\pm0.03$	$1.19^{k}\pm0.04$	$0.64^{a}\pm0.04$	$1.62^{a}\pm0.04$	$0.38^{i}\pm0.04$	$4.26^{a}\pm0.04$
Cs	$0.28^{m}\pm0.02$	$1.47^{bc}\pm0.05$	$0.19^{m}\pm0.05$	$0.60^{l}\pm0.03$	$0.61^{bc}\pm0.03$	$0.98^{l}\pm0.03$
S1 +Cs	$0.33^{j}\pm0.01$	$1.44^{cde}\pm0.04$	$0.23^{j}\pm0.04$	$0.66^{k}\pm0.03$	$0.58^{cd}\pm0.06$	$1.14^{j}\pm0.02$
S2 +Cs	$0.45^{h}\pm0.05$	$1.40^{fg}\pm0.02$	$0.32^{h}\pm0.07$	$0.81^{h}\pm0.06$	$0.55^{de}\pm0.05$	$1.47^{h}\pm0.04$
S3 +Cs	$0.60^{e}\pm0.04$	$1.34^{h}\pm0.06$	$0.45^{e}\pm0.03$	$1.31^{e}\pm0.03$	$0.48^{f}\pm0.04$	$2.73^{e}\pm0.02$
S4 +Cs	$0.74^{b}\pm0.04$	$1.24^{j}\pm0.04$	$0.60^{b}\pm0.04$	$1.58^{b}\pm0.03$	$0.41^{hi}\pm0.05$	$3.67^{b}\pm0.05$
CsNPs	$0.24^{n}\pm0.03$	$1.51^{a}\pm0.05$	$0.16^{n}\pm0.06$	$0.55^{m}\pm0.03$	$0.65^{a}\pm0.04$	$0.85^{m}\pm0.04$
S1 +CsNPs	$0.31^{k}\pm0.03$	$1.49^{ab}\pm0.03$	$0.21^{k}\pm0.04$	$0.61^{l}\pm0.04$	$0.62^{ab}\pm0.03$	$0.98^{l}\pm0.04$
S2 +CsNPs	$0.41^{i}\pm0.06$	$1.45^{cd}\pm0.05$	$0.28^{i}\pm0.04$	$0.77^{i}\pm0.05$	$0.59^{bc}\pm0.03$	$1.31^{i}\pm0.05$
S3 +CsNPs	$0.55^{f}\pm0.05$	$1.41^{ef}\pm0.03$	$0.39^{f}\pm0.02$	$1.26^{f}\pm0.05$	$0.54^{e}\pm0.03$	$2.33^{f}\pm0.05$
S4 +CsNPs	$0.72^{c}\pm0.05$	$1.35^{h}\pm0.03$	$0.53^{c}\pm0.06$	$1.46^{c}\pm0.02$	$0.43^{gh}\pm0.05$	$3.56^{c}\pm0.06$

Means (of three replicates ± standard error) in each column followed by a similar letter are not significantly different at $P \leq 0.05$ using Duncan test.

Supplementary Table S2. Effects of exogenous application of chitosan or chitosan nanoparticles on proline, hydrogen peroxide, lipid peroxidation and electrolyte leakage content of salt-stressed common bean plants at flowering stage grown in clay-sandy soil

Treatments	Proline (μmol/g DW)	H_2O_2 (μmol/g FW)	Lipid peroxidation (μmol MDA/g FW)	Electrolyte leakage (%)
Control	$0.65^{i}\pm0.02$	$18.75^{k}\pm0.20$	$5.90^{ij}\pm0.04$	$57.57^{gh}\pm0.22$
S1	$1.22^{g}\pm0.06$	$20.54^{j}\pm0.43$	$6.42^{fg}\pm0.03$	$59.67^{e}\pm0.33$
S2	$1.32^{fg}\pm0.05$	$38.21^{g}\pm0.32$	$7.35^{d}\pm0.04$	$61.90^{d}\pm0.53$
S3	$1.57^{d}\pm0.04$	$45.11^{d}\pm0.34$	$8.06^{c}\pm0.03$	$65.95^{c}\pm0.23$
S4	$1.78^{c}\pm0.02$	$60.46^{a}\pm0.22$	$10.97^{a}\pm0.04$	$71.72^{a}\pm0.45$
Cs	$0.83^{h}\pm0.03$	$15.05^{m}\pm0.33$	$4.74^{l}\pm0.03$	$56.84^{gh}\pm0.12$
S1 +Cs	$1.38^{ef}\pm0.03$	$16.61^{l}\pm0.13$	$5.97^{hi}\pm0.03$	$57.76^{fg}\pm0.34$
S2 +Cs	$1.45^{e}\pm0.05$	$32.20^{h}\pm0.33$	$5.55^{jk}\pm0.05$	$58.62^{fg}\pm0.35$
S3 +Cs	$1.79^{c}\pm0.03$	$40.11^{e}\pm0.36$	$6.97^{de}\pm0.04$	$60.97^{de}\pm0.33$
S4 +Cs	$1.94^{b}\pm0.04$	$59.68^{b}\pm0.33$	$9.85^{b}\pm0.05$	$69.52^{b}\pm0.32$
CsNPs	$0.87^{h}\pm0.04$	$13.30^{o}\pm0.23$	$4.57^{l}\pm0.05$	$56.17^{hi}\pm0.23$
S1 +CsNPs	$1.44^{ef}\pm0.04$	$14.54^{n}\pm0.12$	$5.46^{k}\pm0.02$	$57.35^{fg}\pm0.44$
S2 +CsNPs	$1.61^{d}\pm0.06$	$30.96^{i}\pm0.43$	$5.21^{k}\pm0.06$	$58.15^{fg}\pm0.36$
S3 +CsNPs	$1.95^{b}\pm0.03$	$38.96^{f}\pm0.40$	$6.35^{gh}\pm0.02$	$59.80^{ef}\pm0.43$
S4 +CsNPs	$2.10^{a}\pm0.03$	$55.98^{c}\pm0.31$	$6.81^{ef}\pm0.04$	$65.06^{c}\pm0.22$

Means (of three replicates ± standard error) in each column followed by a similar letter are not significantly different at $P \leq 0.05$ using Duncan test.

Supplementary Table S3. Summary of the Two-way ANOVA showing the effect of the main factors: chitosan spray and salinity and their interaction on Na^+, K^+ contents and Na^+/K^+ ratio in shoots and roots of salt stressed common bean plants and proline, hydrogen peroxide, lipid peroxidation and electrolyte leakage content at flowering stage.

Variable and source of variation	df	F	*P*		Variable and source of variation	df	F	*P*
**Shoot Na^+**					**Root Na^+/K^+ ratio**			
Chitosan	2	**104.60**	***		Chitosan	2	**7577.60**	***
Salinity	4	**3376.30**	***		Salinity	4	**133239.70**	***
Chitosan × Salinity	8	**4.60**	***		Chitosan × Salinity	8	**532.60**	***
**Shoot K^+**					**Proline**			
Chitosan	2	**320.67**	***		Chitosan	2	**925.52**	***
Salinity	4	**570.58**	***		Salinity	4	**5350.24**	***
Chitosan × Salinity	8	**12.33**	***		Chitosan × Salinity	8	**14.12**	***
**Shoot Na^+/K^+ ratio**					**Hydrogen peroxide**			
Chitosan	2	**178.40**	***		Chitosan	2	**9539.95**	***
Salinity	4	**2502.80**	***		Salinity	4	**205589.78**	***
Chitosan × Salinity	8	**11.15**	***		Chitosan × Salinity	8	**212.50**	***
**Root Na^+**					**Lipid peroxidation**			
Chitosan	2	**396.20**	***		Chitosan	2	**1595.77**	***
Salinity	4	**16220.30**	***		Salinity	4	**2281.34**	***
Chitosan × Salinity	8	**12.20**	***		Chitosan × Salinity	8	**160.57**	***
**Root K^+**					**Electrolyte leakage**			
Chitosan	2	**185.00**	***		Chitosan	2	**6344.41**	***
Salinity	4	**610.70**	***		Salinity	4	**19911.54**	***
Chitosan × Salinity	8	**2.00**	**ns**		Chitosan × Salinity	8	**542.16**	***

*** $P \leq 0.001$, ns= non-significant
